# Crimean-Congo hemorrhagic fever virus protein GP38 from isolate M18-China confers broad immunological breadth

**DOI:** 10.21203/rs.3.rs-9727753/v1

**Published:** 2026-05-19

**Authors:** Albert Wang, Stephanie R. Monticelli, Christy K. Hjorth, Thomas G. Batchelor, Alexandra L. Tse, Ana I. Keuhne, Russell R. Bakken, N. A. Saavedra-Avila, Gorka Lasso, Jacob Berrigan, Steven A. Porcelli, Jason S. McLellan, Andrew S. Herbert, Kartik Chandran

**Affiliations:** Albert Einstein College of Medicine; U.S. Army Medical Research Institute of Infectious Diseases; The University of Texas at Austin; U.S. Army Medical Research Institute of Infectious Diseases; Albert Einstein College of Medicine; U.S. Army Medical Research Institute of Infectious Diseases; U.S. Army Medical Research Institute of Infectious Diseases; Albert Einstein College of Medicine; Albert Einstein College of Medicine; Albert Einstein College of Medicine; Albert Einstein College of Medicine; The University of Texas at Austin; U.S. Army Medical Research Institute of Infectious Diseases; Albert Einstein College of Medicine

## Abstract

Crimean-Congo hemorrhagic fever virus (CCHFV) is a tick-borne, negative-strand RNA virus that causes outbreaks of lethal hemorrhagic fever. No approved vaccines or specific antiviral treatments are available. CCHFV glycoprotein GP38 is an integral component of the pre-fusion glycoprotein entry complex, plays roles in viral pathogenesis, and is a key target of antibody-mediated protection. Herein, we investigated the hypothesis that recombinant GP38 itself could elicit protection against divergent CCHFV isolates/strains in an animal model. Accordingly, we generated GP38 immunogens from six CCHFV clades and showed that GP38 from the isolate M18-China induces a broad immunological response in mice despite its lower amino acid sequence similarity to other isolates. We identified sequences in M18-China GP38 associated with this immunological breadth and evaluated it as a vaccine candidate. Although M18-China GP38 was not protective in isolation, our findings warrant further exploration of its utility as one component of a broadly protective CCHFV vaccine.

## INTRODUCTION

Crimean-Congo hemorrhagic fever virus (CCHFV) is a high-priority human pathogen causing hemorrhage with an average case fatality rate of 30%^1,2^. Spread primarily by the *Hyalomma* species of ticks, CCHFV is transmitted to animals and livestock often found in contact with humans^3–5^. Correspondingly, CCHF outbreaks occur in over 30 countries, and annual incidence has been steadily rising worldwide due to increased human migration and host tick range expansion from ecological changes^5,6^. This widespread geographic distribution results in substantial genetic diversity that translates to differences in virulence, pathogenicity, and clinical significance in humans^1,7^. There are no approved vaccines or antiviral therapeutics to treat CCHFV beyond supportive care^1,2,8^. Consequently, there is an urgent need to develop such countermeasures.

One of the challenges in vaccinating against CCHFV is the virus’s significant genetic diversity. The CCHFV genome comprises three single-stranded RNA genome segments: small (S), medium (M), and large (L), similar to other *Bunyaviricetes* members^1,2,9^. The S, M, and L segments respectively encode the nucleoprotein (NP) and non-structural protein NSs, the glycoprotein precursor complex (GPC), and the RNA-dependent RNA polymerase (RdRP). Genetic analyses on the CCHFV genome segments have indicated that the NP and RdRP proteins have high amino acid sequence conservation across viral isolates (> 95%); conversely, the GPC has relatively low sequence conservation (< 75% in divergent isolates)^2^. The GPC polyprotein is proteolytically processed in host cells to yield the structural glycoproteins Gn and Gc, the nonstructural protein NSm, and the mucin-like domain (MLD), GP160/85, and GP38. Previous vaccine efforts have largely targeted Gc or the entire M segment, as Gn and Gc are present on the surface of the CCHFV particle and mediate virus binding and entry^8–12^. GP38 is also an important vaccine target due to its roles in Gc pre-fusion stabilization, pathogenesis, and vascular leak. Since its discovery, GP38 has been characterized as a secreted glycoprotein, lacking transmembrane domains and found only in virus-infected cellular supernatant^13^. However, the structural characterization of an engineered and disulfide-bond stabilized heterotrimeric GPC complex revealed that GP38 is an integral constituent of the pre-fusion GPC entry complex and that GP38 and Gn together likely chaperone Gc in its pre-fusion state^14^. Further, the murine antibody 13G8 and several human-derived GP38-targeting monoclonal antibodies (mAbs) have been shown to partially protect against lethal infection by several divergent CCHFV isolates in rodents, demonstrating the therapeutic potential of targeting GP38^15–18^. More recent work suggests that GP38 is a viral toxin that induces endothelial permeability *in vitro* and vascular leak *in vivo*^19^. Therefore, a GP38-based vaccine could target and block the function of GP38 in both its virus-associated and soluble forms.

Concurrent developments of other GP38-based vaccines have indicated that this approach may be viable. Mice vaccinated with an inactivated rabies virus that expressed GP38 were 100% protected against lethal CCHFV infection^20^. Further, vaccine efficacy has been shown to decrease in the absence of GP38. Specifically, Suschak et al. (2021) showed that vaccinating mice with a M-segment-encoding DNA vaccine led to 100% protection against CCHFV. However, in the absence of GP38, they observed a complete loss in protection^21^. Historically, CCHFV vaccine development has focused on protecting against homologous challenge, but more recent work has emphasized the importance of developing vaccines capable of broadly protecting against multiple, genetically diverse isolates^7,21–24^. Herein, we assess the potential for immunogenicity differences across GP38 isolates and apply our findings to target CCHFV breadth in the context of vaccination. We generated six recombinant GP38s (rGP38s) chosen from across the phylogenetic landscape and assessed serum reactivity to cognate or divergent GP38s. Despite the high sequence conservation of GP38 sequences, we found that GP38 from two CCHFV isolates, M18-China and IbAr10200, induced broadly cross-reactive antibody responses in mice, whereas other GP38s failed to do so. M18-China-rGP38 elicited an antibody response that was exceptional in its strength and pan-CCHFV clade breadth. We further identified the GP38 sequences that are likely responsible for this broad antibody response. Finally, we assessed the extent to which vaccination with GP38 derived from M18-China or IbAr10200 in both subunit and VSV-based vaccine formats confers protection against CCHFV challenge in mice.

## Results

### Phylogenetic analysis of CCHFV GP38 shows 6 major branches

**Figure 1:**
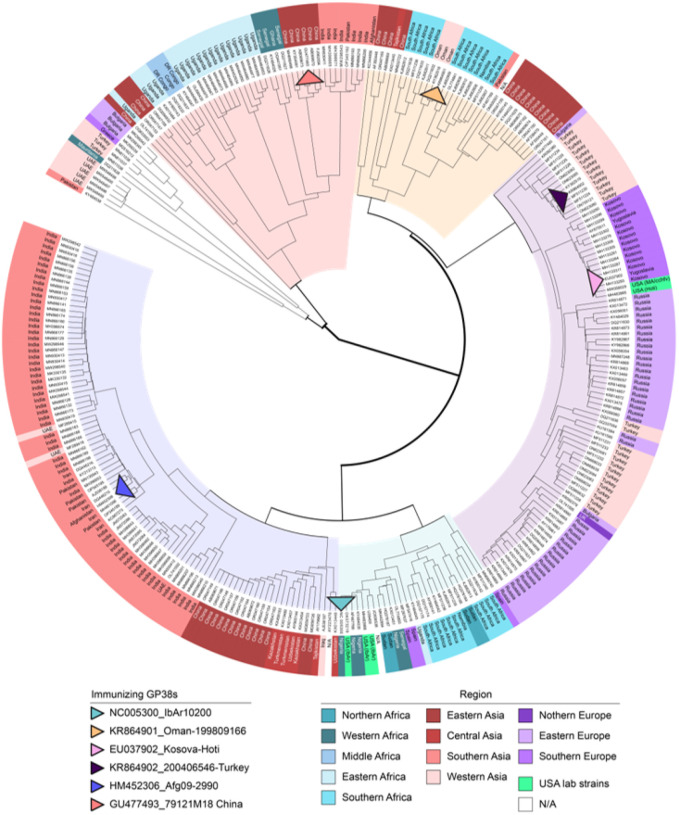
CCHFV-GP38 phylogenetic tree shows 6 major branches Amino-acid based tree depicting 302 unique CCHFV-GP38 proteins. Colored ranges denote different clades. The outer ring is color-coded according to the region where CCHFV was isolated. Each GP38 protein is labelled with its corresponding GenBank ID. Colored arrowheads indicate representative GP38 proteins used in subsequent immunization studies.

We performed a multiple sequence alignment (MSA) on 302 available GP38 amino acid sequences from GenBank and generated a tree to describe the evolutionary relationships between diverse CCHFV GP38 proteins (Fig. 1). The tree showed two large branches that were further subdivided into six separate clades. Previous work has utilized the full M genome segment to reconstruct the evolutionary history of CCHFV and reported six or seven clades^1,25–27^. We did not find 1:1 correspondence between the two sets of clades, likely reflecting that each tree is based on different information content (e.g., GP38-only amino acids versus the entire GPC polyprotein M-segment nucleotide sequence). Six representative GP38 sequences corresponding to six available authentic virus stocks were chosen across the phylogenetic tree and further investigated for immunogenicity (Fig. 1).

Amino-acid based tree depicting 302 unique CCHFV-GP38 proteins. Colored ranges denote different clades. The outer ring is color-coded according to the region where CCHFV was isolated. Each GP38 protein is labelled with its corresponding GenBank ID. Colored arrowheads indicate representative GP38 proteins used in subsequent immunization studies.

#### Recombinant GP38 derived from M18-China induces stronger homotypic and heterotypic responses than other GP38 isolates in mice

**Figure 2:**
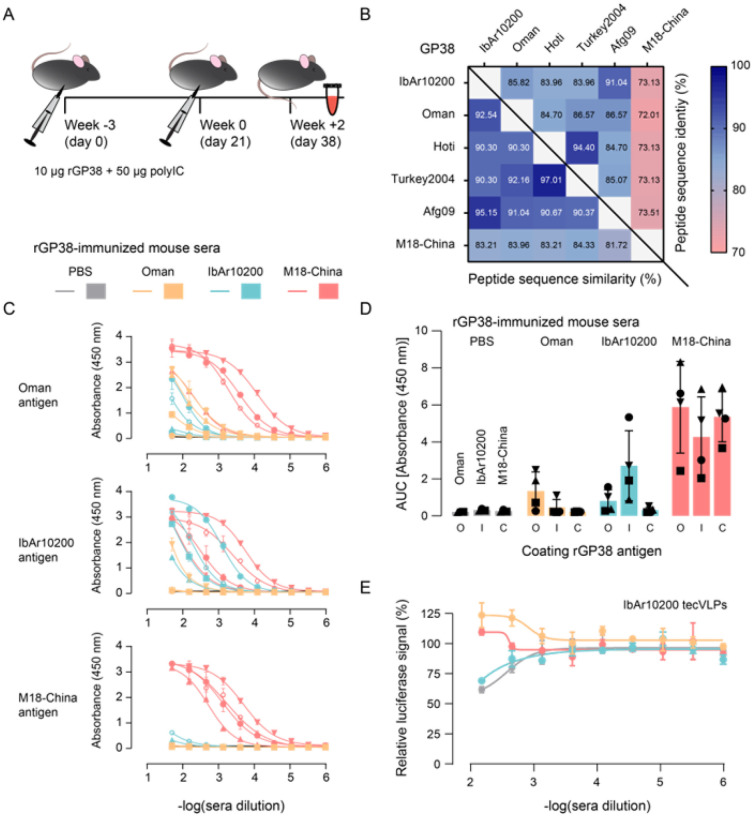
CCHFV-GP38 exhibits strain-specific immunogenicity **2A** Schematic of mouse immunization: 10 μg of each rGP38 (Oman, IbAr10200, M18-China) adjuvanted with 50 μg polyIC were injected into female C57BL/6J mice (n=5) i.p. prime and boost. Sera was collected +2 weeks post-boost and analyzed. **2B** Amino acid sequence similarity (polarity, charge) and identity across GP38 isolates (calculated by the Sequence Manipulation Suite^30^). **2C** rGP38-immunized mouse sera ELISAs, homotypic and heterotypic, against Oman, IbAr10200, and M18-China GP38 antigen (N=1, n=2). Mouse sera within each group are individually plotted and delineated by separate shapes. **2D** Corresponding area under curve values were calculated for each serum sample ELISA; the mean value is plotted. **2E** Sera from each immunization group was pooled and evaluated for neutralization against tecVLPs (N=1, n=3). Infectivity was normalized to no sera control. Exact p-values and descriptive statistics values can be found in **Methods**. Error bars indicate standard deviation.

Six rGP38s were expressed with their respective mucin-like domains (MLDs), cleaved, purified, and shown to bind to conformationally sensitive GP38-specific mAbs, ADI-58048 and ADI-63530^18^, in enzyme-linked immunosorbent assays (ELISAs) (**Supplemental Fig. 1A-D**). To examine the immunogenicity elicited by each rGP38, we immunized female C57BL/6J mice (n = 5 per group) with two doses, 3-weeks apart, of each rGP38 (10 μg) in combination with polyinosinic:polycytidylic acid (polyIC, 50 μg) by the intraperitoneal route (i.p.) (Fig. 2A). We chose to first investigate representative rGP38s from each large branch of the GP38 phylogenetic tree: Oman, IbAr10200, and M18-China. Analysis of the peptide sequence of each GP38 revealed that the CCHFV-M18-China GP38 was the most divergent in this group (Fig. 2B). We thus hypothesized that IbAr10200- and Oman-rGP38 immunogens would induce a better cross-reactive response to each other than that of M18-China. Two weeks after boost, sera were isolated and evaluated for homotypic and heterotypic GP38 binding by ELISA (Fig. 2C), and area under the curve (AUC) values were calculated (Fig. 2D). As expected, phosphate-buffered saline (PBS)-immunized sera (PBS-sera) showed little or no reactivity to any rGP38. Contrary to our expectations, we observed isolate-dependent differences in binding. Oman-rGP38-immunized sera (Oman-sera) bound the Oman-rGP38 antigen (homotypic response), as expected, but responded poorly against IbAr10200- or M18-China-rGP38 antigens (heterotypic responses). IbAr10200-rGP38-immunized sera (IbAr10200-sera) showed a strong homotypic response but weak heterotypic responses (some cross reactivity to Oman-rGP38 but no detectable binding to M18-China-rGP38). By contrast, sera immunized with rGP38 from the M18-China isolate (M18-China-sera) showed both homotypic and heterotypic responses. For example, 60% of the M18-China-sera samples bound more strongly to Oman-rGP38 antigen than did the cognate Oman-sera. None of the sera neutralized infection by the CCHFV model system of transcription- and entry-competent virus-like particles (tecVLPs) (Fig. 2E), as expected—the lack of neutralization by GP38-targeting antibodies is well-documented^15,17,18,28,29^.

**2A** Schematic of mouse immunization: 10 μg of each rGP38 (Oman, IbAr10200, M18-China) adjuvanted with 50 μg polyIC were injected into female C57BL/6J mice (n = 5) i.p. prime and boost. Sera was collected + 2 weeks post-boost and analyzed. **2B** Amino acid sequence similarity (polarity, charge) and identity across GP38 isolates (calculated by the Sequence Manipulation Suite^30^). **2C** rGP38-immunized mouse sera ELISAs, homotypic and heterotypic, against Oman, IbAr10200, and M18-China GP38 antigen (N = 1, n = 2). Mouse sera within each group are individually plotted and delineated by separate shapes. **2D** Corresponding area under curve values were calculated for each serum sample ELISA; the mean value is plotted. **2E** Sera from each immunization group was pooled and evaluated for neutralization against tecVLPs (N = 1, n = 3). Infectivity was normalized to no sera control. Exact p-values and descriptive statistics values can be found in **Methods**. Error bars indicate standard deviation.

### M18-China rGP38 induces a broad immunological response up to 14 weeks post-vaccination in mice

**Figure 3:**
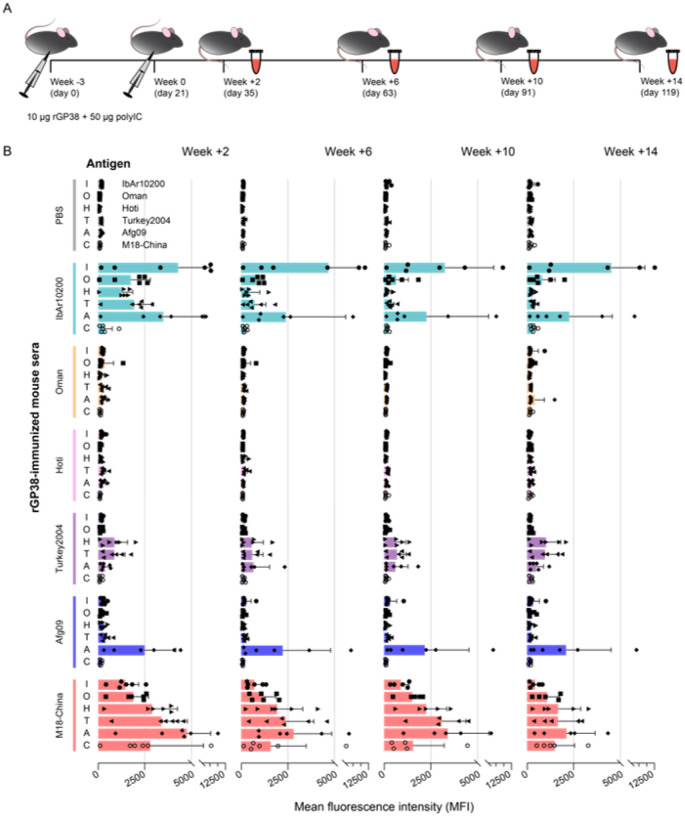
M18-China rGP38 induces a durable, cross-reactive response in mice **3A** Schematic of mouse immunization (n=6; 10 μg rGP38 [IbAr10200, Oman, Hoti, Turkey2004, Afg09, M18-China] or PBS + 50 μg polyIC i.p) and sera collection schedule. **3B** Binding of rGP38-immunized sera to antigen (rGP38)-coupled microspheres by MAGPIX. Each point represents the average of two experiments where each serum sample was simultaneously incubated with all antigens (N=2, n=4). Sera groups are delineated by different colors (left-border) while antigens are indicated by lettering and shape (right-border). Error bars indicate standard deviation. Descriptive statistics values can be found in **Methods**.

We sought to repeat this finding and evaluate a larger number of GP38 proteins by re-immunizing a new set of C57BL/6J mice (n = 6, prime and boost) with all six purified rGP38 proteins (10 μg; IbAr10200, Oman, Hoti, Turkey2004, Afg09, M18-China) or PBS adjuvanted with polyIC (50 μg). We also extended the study period to 14 weeks following the 2nd dose to assess the durability of the immunological response. Mouse sera were isolated every 4 weeks starting from + 2 weeks post-boost (Fig. 3A). We evaluated homotypic and heterotypic antigen binding to all six antigens using MAGPIX. M18-China-sera (red) bound to all rGP38 antigens, replicating and extending the findings in our previous study (Fig. 3B). This extraordinary breadth was observed across all timepoints sampled; however, M18-China-sera reactivity against IbAr10200-rGP38 was mostly lost after + 14 weeks. IbAr10200-sera bound to all rGP38 antigens except against M18-China-GP38 + 2 weeks. The sera also reacted well to Afg09-rGP38, consistent with the GPC polyprotein^1^ and GP38 sequences of IbAr10200 and Afg09 clustering together (Fig. 1). The IbAr10200-sera (aqua) heterotypic responses to rGP38 antigens waned over time, with some mice showing a response to only Oman- and Afg09-rGP38 by + 14 weeks post-boost. Unexpectedly, minimal binding of Hoti-rGP38-immunized sera (Hoti-sera, pink) and Oman-sera (orange) was observed for any rGP38, including the cognate antigens. Approximately 50% of the mice immunized with Turkey2004-rGP38 (purple) showed a homotypic response. Sera from these responding mice could further cross-react against Hoti- and Afg09-rGP38. Lastly, Afg09-rGP38-immunized mouse sera (Afg09-sera; indigo) only bound to its cognate antigen, and interestingly, could not reciprocally recognize IbAr10200-rGP38.

**3A** Schematic of mouse immunization (n = 6; 10 μg rGP38 [IbAr10200, Oman, Hoti, Turkey2004, Afg09, M18-China] or PBS + 50 μg polyIC i.p) and sera collection schedule. **3B** Binding of rGP38-immunized sera to antigen (rGP38)-coupled microspheres by MAGPIX. Each point represents the average of two experiments where each serum sample was simultaneously incubated with all antigens (N = 2, n = 4). Sera groups are delineated by different colors (left-border) while antigens are indicated by lettering and shape (right-border). Error bars indicate standard deviation. Descriptive statistics values can be found in **Methods**.

#### The alpha-helices, variable loop, and extended loop of GP38 contribute to the broad cross-reactivity induced by M18-China rGP38

**Figure 4:**
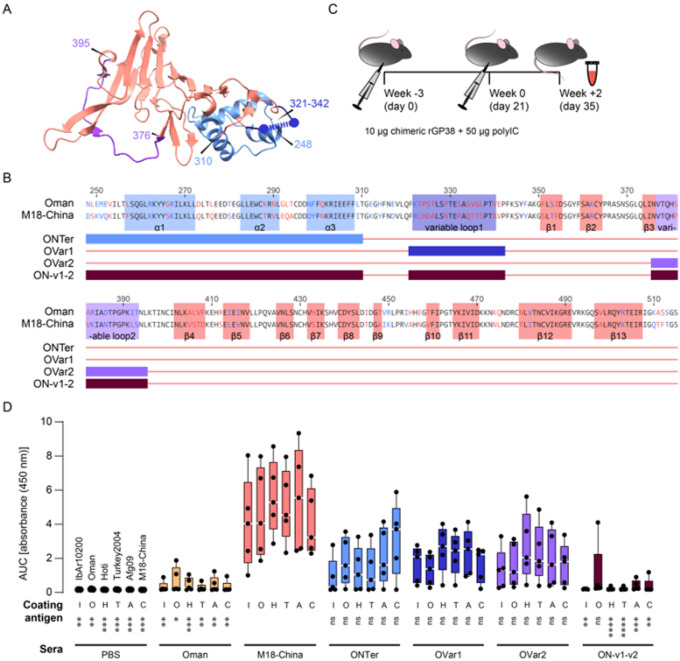
GP38 α-helices, variable, and extended loop confer broad immunogenicity **4A** CCHFV-IbAr10200 GP38 (PDB: 6VKF, Mishra et al., 2020^16^) with chimerized regions in light blue, blue, or purple on the red structure (residues indicated). OVar1 GP38 encompasses a substitution of an uncrystallized region (dashed line). **4B** Sequence alignment of GP38 from CCHFV-Oman and -M18-China strains with major regions labeled in accordance with Shin, Monticelli, Hjorth et al. (2024)^18^. Chimeras are composed of inserting the Oman GP38 sequences into M18-China GP38. Identical, similar, and dissimilar amino acid residues are colored black, blue, and red, respectively. **4C** Mouse immunization schematic (n=5) of rGP38 (Oman, M18-China), chimeric rGP38 (ONTer, OVar1, OVar2, and ON-v1–2), and PBS. **4D** Heterotypic chimeric rGP38-immunized mouse sera ELISAs: AUC values of each sera group against IbAr10200, Oman, Hoti, Turkey2004, Afg09, and M18-China rGP38 antigen (N=1, n=2) are individually plotted (5 mice / group). Box-and-whisker plots of AUC values depict range, quartile, and median (white line). AUC values were log_10_-transformed and compared by repeated-measure 2-way ANOVA with Dunnett’s multiple comparisons test where sera response was compared against that of M18-China-sera for each coating antigen. ns - not significant; * p <0.05; ** p < 0.01; *** p <0.001; **** p<0.0001. Exact p-values and descriptive statistics values can be found in **Methods.**

We asked why M18-China-rGP38 was more immunogenic than the other GP38s tested herein. Although IbAr10200-sera was broadly cross-reactive, it could not bind to M18-China-rGP38 (Figs. 2D, 3B). Previous sequence analyses of the six GP38s used in this study revealed that the variable loop (amino acid residues 322–340) is poorly conserved^18^. This region is highly flexible and disordered in a GP38 X-ray crystal structure^16^ (Fig. 4A). We thus hypothesized that this region of M18-China-GP38 may be responsible for its broad immunogenicity. Accordingly, we designed, expressed, and evaluated a panel of Oman-China rGP38 chimeras (Fig. 4B). We chose to chimerize the Oman and China rGP38 proteins because of their starkly different capacities to elicit a heterotypic antibody response—weak for Oman and strong for China (Fig. 2–3). First, we substituted the variable loop from Oman-GP38 into M18-China-GP38, termed OVar1. Noting additional sequences that showed divergence, we next substituted the Oman α-helical loops in the N-terminus or extended loop (hereon referred to as variable loop 2) into M18-China GP38, yielding GP38 molecules ONTer and OVar2. Finally, we incorporated all three Oman substitutions into M18-China to generate the chimeric molecule ON-v1–2. We postulated that substitution of a highly immunogenic sequence in M18-China-rGP38 with its poorly immunogenic Oman counterpart should reduce the capacity of the chimera to elicit heterotypic GP38 responses (i.e., “loss-of-function”). The chimeric GP38 molecules were expressed with M18-China MLD, cleaved, purified, and tested for proper folding with conformation-sensitive mAbs ADI-58048 and ADI-63530 (**Supplementary Fig. 2A-D**) prior to immunization.

Female C57BL/6J mice (n = 5) were injected i.p. with the same 2-dose immunization regimen of rGP38 (10 μg; Oman, M18-China, OVar1, OVar2, ONTer, ON-v1–2) or PBS adjuvanted with polyIC (50 μg), and sera were isolated + 2 weeks post-boost (Fig. 4C). Homotypic antigen ELISAs showed that 3–4 mice responded well in the ONTer-, OVar1-, and OVar2-immunized groups. However, only a single mouse in the ON-v1–2 group displayed a similar reactivity (**Supplementary Fig. 3A**), indicating that replacing all three regions of GP38 with sequences from the Oman isolate decreased its immunogenicity. Heterologous rGP38 ELISAs with Oman- and M18-China-sera confirmed our previous findings of cross-reactivity (Fig. 4D, **Supplementary Fig. 3B**). By ELISA, sera from ON-v1–2-vaccinated mice were significantly less reactive than M18-China-sera to five of the six rGP38s in this study, including to M18-China-rGP38. Whereas binding to Oman-rGP38 was not statistically different between ON-v1–2- and M18-China-sera, only one mouse in the ON-v1–2-rGP38 immunization group showed comparable binding. ONTer-, OVar1-, and OVar2-rGP38-immunized mouse sera all showed a decrease in reactivity against each of the six rGP38s as compared to M18-China-sera (Fig. 4D). Therefore, all three sequences in GP38—the N-terminal alpha-helices, variable loop, and extended loop—together contribute to the elicitation of cross-reactive antibodies.

**4A** CCHFV-IbAr10200 GP38 (PDB: 6VKF, Mishra et al., 2020^16^) with chimerized regions in light blue, blue, or purple on the red structure (residues indicated). OVar1 GP38 encompasses a substitution of an uncrystallized region (dashed line). **4B** Sequence alignment of GP38 from CCHFV-Oman and -M18-China strains with major regions labeled in accordance with Shin, Monticelli, Hjorth et al. (2024)^18^. Chimeras are composed of inserting the Oman GP38 sequences into M18-China GP38. Identical, similar, and dissimilar amino acid residues are colored black, blue, and red, respectively. **4C** Mouse immunization schematic (n = 5) of rGP38 (Oman, M18-China), chimeric rGP38 (ONTer, OVar1, OVar2, and ON-v1–2), and PBS. **4D** Heterotypic chimeric rGP38-immunized mouse sera ELISAs: AUC values of each sera group against IbAr10200, Oman, Hoti, Turkey2004, Afg09, and M18-China rGP38 antigen (N = 1, n = 2) are individually plotted (5 mice / group). Box-and-whisker plots of AUC values depict range, quartile, and median (white line). AUC values were log_10_-transformed and compared by repeated-measure 2-way ANOVA with Dunnett’s multiple comparisons test where sera response was compared against that of M18-China-sera for each coating antigen. ns - not significant; * p < 0.05; ** p < 0.01; *** p < 0.001; **** p < 0.0001. Exact p-values and descriptive statistics values can be found in **Methods**.

### Recombinant GP38 subunit vaccination does not provide significant protection against CCHFV infection in mice

**Figure 5:**
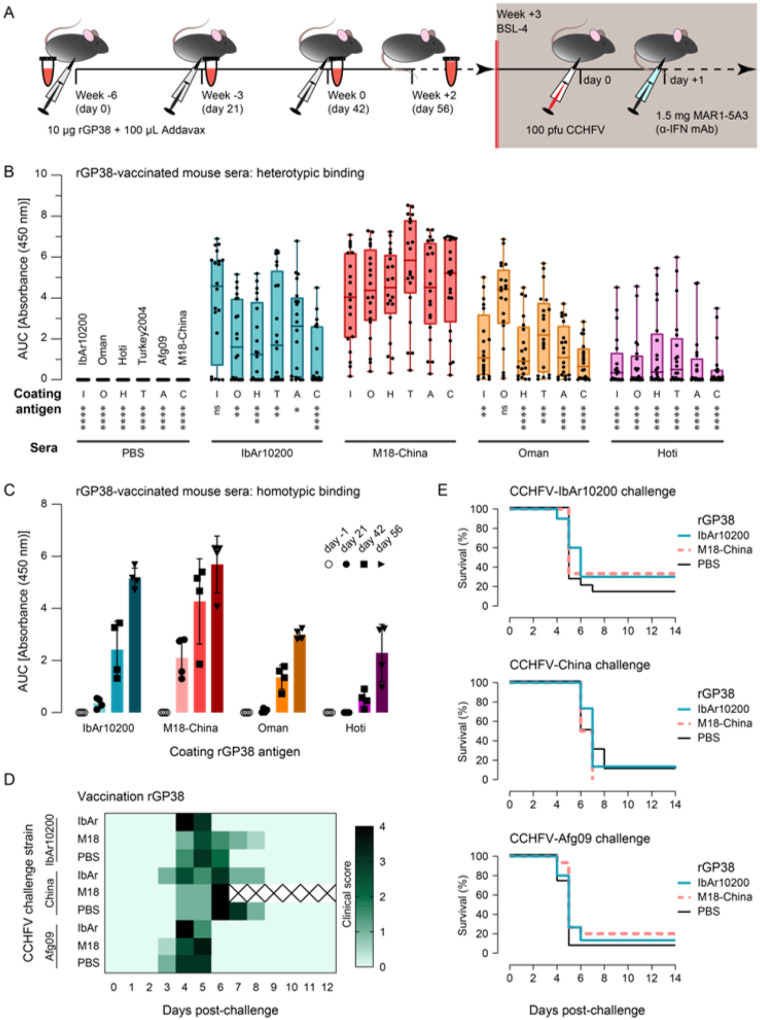
IbAr10200- and M18-China-rGP38 vaccination do not significantly protect against lethal CCHFV challenge in mice **5A** Schematic of mouse vaccination (n=20; 10 μg rGP38 [IbAr10200, Oman, Hoti, M18-China] or PBS + Addavax i.p), sera collection schedule, and CCHFV challenge. After vaccination, mice were challenged with 100 pfu CCHFV-IbAr10200, -Afg09, or China and treated with 1.5 mg of MAR1–5A3 antibody. **5B** Heterotypic rGP38-vaccinated mouse sera (day 56) ELISAs: AUC values of each sera group against IbAr10200, Oman, Hoti, Turkey2004, Afg09, and M18-China rGP38 antigen (N=1, n=2) are individually plotted (20 mice / group). Box-and-whisker plots of AUC values depict range, quartile, and median. AUC values were compared by repeated-measure 2-way ANOVA with Dunnett’s multiple comparisons test where sera response was compared against that of M18-China-sera for each coating antigen. **5C** Homotypic rGP38-vaccinated mouse sera ELISAs: AUC values of each sera group against their self-antigens (N=1, n=2) are plotted over time relative to prime immunization (day −1, 21, 42, 56). Each point represents the pooling of 5 sera samples per group. Error bars are indicative of standard deviation. **5D** Clinical scores of rGP38-vaccinated mice challenged against indicated CCHFV isolate. **5E** Survival curves of IbAr10200 rGP38-, M18-China rGP38- or PBS-vaccinated mice challenged against CCHFV-IbAr10200, CCHFV-Afg09, or CCHFV-China (n=10–15). Survival for each vaccination condition was compared to control (PBS) by logrank test. All comparisons were not significant. ns - not significant; * p <0.05; ** p < 0.01; *** p <0.001; **** p<0.0001. Exact p-values and descriptive statistics values can be found in **Methods**.

We sought to extend cross-reactivity to cross-protection. Previous studies have shown that vaccinating C57BL/6J mice with IbAr10200-rGP38 in an immunosuppression model can confer approximately 40% protection against a homologous CCHFV-IbAr10200 challenge^14,31^. Based on our immunogenicity results that demonstrate greater cross-reactivity and duration of humoral immunity, we hypothesized that vaccination with M18-China rGP38 would result in improved protective efficacy in this model. Mice were immunized i.p. three times on days 0, 21, and 42 with rGP38 (10 μg) from IbAr10200, M18-China, Oman, and Hoti isolates adjuvanted with Addavax (Fig. 5A). Days 0, 21, 42, and 56 post-prime immunization sera were isolated and evaluated for homotypic and heterotypic reactivity to our rGP38 panel by ELISA (**Supplementary Fig. 4A-B**), and AUC values were calculated for each sample (Fig. 5B–C). As observed previously, M18-China-sera bound significantly better to nearly all rGP38 antigens as compared to IbAr10200-, Oman-, Hoti-, or PBS-sera (Fig. 5B). The only conditions where similar binding occurred were the homotypic reactivity of IbAr10200- or Oman-sera to their cognate antigens as compared to M18-China-sera cross-reactivity. With three vaccination doses, IbAr10200-sera and Oman-sera responded better. A few of the IbAr10200-sera samples could react to M18-China-rGP38, but most still bound poorly. By contrast, Oman-sera showed broad cross-reactivity (even to M18-China GP38) and bound Turkey2004-GP38 as well as IbAr10200-sera. Interestingly, while some mice responded to Hoti-rGP38 vaccination, most of the Hoti-sera samples still responded poorly to all GP38 antigens.

When we compared sera reactivity to their cognate antigens over time, we noticed that different rGP38s induced different immunological response times. Mice immunized with M18-China-rGP38 responded the earliest, followed by groups immunized with the IbAr10200-, Oman-, and Hoti-rGP38s (Fig. 5C, **Supplementary Fig. 4B**). Notably, the response by M18-China-sera after the first dose was equivalent to or greater than that of other groups that had received two (IbAr10200, Oman) or even three doses (Hoti).

Mice immunized with rGP38 from IbAr10200 or M18-China or PBS vehicle were subsequently challenged with the authentic CCHFV isolates IbAr10200, Afg09, or China + 3 weeks post-final vaccination to assess broad protective efficacy. Mice were transiently immunosuppressed with 1.5 mg per mouse of MAR1–5A3 antibody 24 hours (h) post-challenge. All mice exhibited rapid weight loss and registered high clinical scores 4–6 days after challenge (Fig. 5D, **Supplemental Fig. 4C**). None of the rGP38-vaccinated mice showed statistically significantly better survival as compared to the controls for homologous or heterologous CCHFV challenge (Fig. 5E). rGP38-immunized mice challenged with the CCHFV strain IbAr10200 had the greatest survival at 30%.

**5A** Schematic of mouse vaccination (n = 20; 10 μg rGP38 [IbAr10200, Oman, Hoti, M18-China] or PBS + Addavax i.p), sera collection schedule, and CCHFV challenge. After vaccination, mice were challenged with 100 pfu CCHFV-IbAr10200, -Afg09, or China and treated with 1.5 mg of MAR1–5A3 antibody. **5B** Heterotypic rGP38-vaccinated mouse sera (day 56) ELISAs: AUC values of each sera group against IbAr10200, Oman, Hoti, Turkey2004, Afg09, and M18-China rGP38 antigen (N = 1, n = 2) are individually plotted (20 mice / group). Box-and-whisker plots of AUC values depict range, quartile, and median. AUC values were compared by repeated-measure 2-way ANOVA with Dunnett’s multiple comparisons test where sera response was compared against that of M18-China-sera for each coating antigen. **5C** Homotypic rGP38-vaccinated mouse sera ELISAs: AUC values of each sera group against their self-antigens (N = 1, n = 2) are plotted over time relative to prime immunization (day – 1, 21, 42, 56). Each point represents the pooling of 5 sera samples per group. Error bars are indicative of standard deviation. **5D** Clinical scores of rGP38-vaccinated mice challenged against indicated CCHFV isolate. **5E** Survival curves of IbAr10200 rGP38-, M18-China rGP38- or PBS-vaccinated mice challenged against CCHFV-IbAr10200, CCHFV-Afg09, or CCHFV-China (n = 10–15). Survival for each vaccination condition was compared to control (PBS) by logrank test. All comparisons were not significant. ns - not significant; * p < 0.05; ** p < 0.01; *** p < 0.001; **** p < 0.0001. Exact p-values and descriptive statistics values can be found in **Methods**.

### Alternative vectors for GP38 may improve antigen presentation and subsequent protection

Two vaccine studies that have demonstrated the viability and importance of using GP38 are the M-segment-based DNA vaccine and the GP38-tethered rhabdovirus-based vaccine^20,31^. We applied our findings to the recombinant vesicular stomatitis virus (rVSV) platform due to its demonstrated history of use as an experimental vaccine for numerous viral pathogens with several advantages, including its ability to grow to high titers, capacity to induce both humoral and T-cell immune responses, and its documented safety profile in animals and humans^32–40^. Furthermore, a rVSV-based vaccine that replaces the native glycoprotein with CCHFV GPC was shown to confer 100% protection in mice after a single vaccination dose^22^.

**Figure 6:**
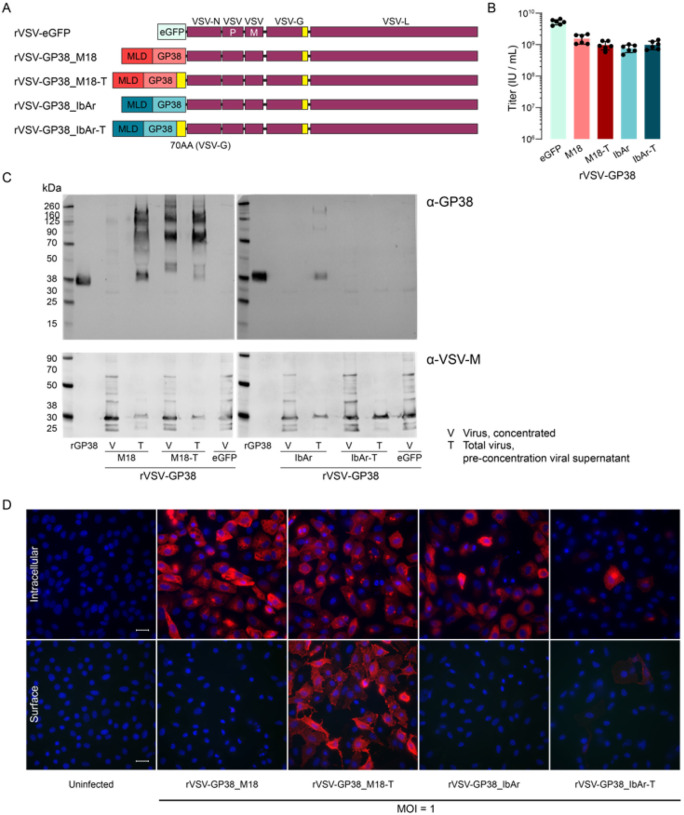
rVSV-GP38_M18-T-infected cells show high levels of GP38 expression **6A** rVSV-GP38 constructs: GP38s are tethered to rVSV by the addition of 70AA from the C-terminus of VSV-G. All constructs are replication-competent. **6B** rVSV-GP38 viruses were titered on Vero cells (N=2, n=6): cells were infected for 1 h before infection was stopped with the addition of 20 mM NH_4_Cl. Error bars indicate standard deviation. **6C** Western blot of concentrated rVSV-GP38 virus and total virus. Equal volume amounts of viral pellet or total virus (pre-concentration viral supernatant) were loaded and run on a non-reducing gel. GP38 and VSV-M were detected by ADI-46152, a human anti-GP38 mAb as described in Shin, Monticelli, Hjorth et al., 2024^18^, and by 23H12, a mouse anti-VSV-M mAb, respectively. **6D** Intracellular and surface staining for GP38 in rVSV-GP38-infected cells. Cells were infected (MOI = 1) for 1 h before the addition of NH_4_Cl. GP38 was detected by ADI-46152 while the nucleus was labeled with Hoescht 33342. White scale bar = 20 μm.

Utilizing an rVSV-eGFP construct containing a gene encoding an enhanced green fluorescent protein (eGFP) sequence before the first native VSV gene, we generated five rVSV genome constructs with two variants for each GP38 strain, IbAr10200 and M18-China (Fig. 6A). In the first variant, we substituted eGFP with the MLD and GP38 (rVSV-GP38_M18, rVSV-GP38_IbAr). We included the MLD to increase GP38 expression^20^. In the second variant, we included the last 70 amino acids of VSV-glycoprotein (VSV-G) to attach GP38 to the surface of the resulting virus particle^41^. We termed these viruses “tethered (T)” viruses (rVSV-GP38_M18-T, rVSV-GP38_IbAr-T). We used a plasmid-based transfection system^42–44^ to rescue each of the five viruses. The resulting rVSV-GP38 viruses showed titers in the same order of magnitude (~ 5×10^8^–1×10^9^ infectious units-per-mL [IU/mL]) (Fig. 6B).

#### Tethering M18-China GP38 to rVSV causes robust intracellular and surface expression of GP38 in infected cells

We next analyzed the level of GP38 expression on the pre-concentrated viral supernatant (total virus) and viral pellets (Fig. 6C). GP38 was undetectable in rVSV-eGFP viral pellets and in the viral pellets of the untethered viruses rVSV-GP38_M18 and rVSV-GP38_IbAr. However, GP38 and higher-order GP38 structures (likely GP85/GP160GP160/GP85) were detected in the total virus, indicating that GP38 was being expressed. This observation was further accentuated in the tethered viruses. GP38 was easily detectable in both the viral pellet and supernatant of rVSV-GP38_M18-T; an approximately 8 kDa increase was observed for the tethered GP38 relative to the GP38 detected in the supernatant or the pellet of the untethered virus. In contrast, no GP38 or GP38-derivatives were detectable in the pellet or supernatant of rVSV-GP38_IbAr-T, despite similar loading amounts, as qualified by VSV-matrix (VSV-M) protein levels.

We infected Vero cells with each rVSV-GP38 (multiplicity of infection [MOI] = 1) for 1 h, stopped infection, and performed intracellular and surface staining for GP38. We observed intracellular GP38 expression throughout the cells infected with rVSV-GP38_M18 and to a lesser extent, in rVSV-GP38_IbAr-infected cells (Fig. 6D). Cells infected with the tethered rVSV-GP38_M18-T also showed a cytoplasmic-wide distribution of GP38 with intense signals clustering near the nucleus. GP38 expression in cells infected with the tethered rVSV bearing IbAr10200 GP38 was low and almost undetectable; in cells that did express GP38, GP38 was also clustered towards the nucleus. On the surface of rVSV-GP38_M18-T-infected cells, we observed robust expression of GP38 in discrete clusters. In contrast, GP38 staining was barely detectable at the same exposure at the surface of rVSV-GP38_IbAr-T-infected cells.

**6A** rVSV-GP38 constructs: GP38s are tethered to rVSV by the addition of 70AA from the C-terminus of VSV-G. All constructs are replication-competent. **6B** rVSV-GP38 viruses were titered on Vero cells (N = 2, n = 6): cells were infected for 1 h before infection was stopped with the addition of 20 mM NH_4_Cl. Error bars indicate standard deviation. **6C** Western blot of concentrated rVSV-GP38 virus and total virus. Equal volume amounts of viral pellet or total virus (pre-concentration viral supernatant) were loaded and run on a non-reducing gel. GP38 and VSV-M were detected by ADI-46152, a human anti-GP38 mAb as described in Shin, Monticelli, Hjorth et al., 2024^18^, and by 23H12, a mouse anti-VSV-M mAb, respectively. **6D** Intracellular and surface staining for GP38 in rVSV-GP38-infected cells. Cells were infected (MOI = 1) for 1 h before the addition of NH_4_Cl. GP38 was detected by ADI-46152 while the nucleus was labeled with Hoescht 33342. White scale bar = 20 μm.

#### Vaccination with rVSV-GP38_M18-T does not provide significant cross-protection in mice.

**Figure 7:**
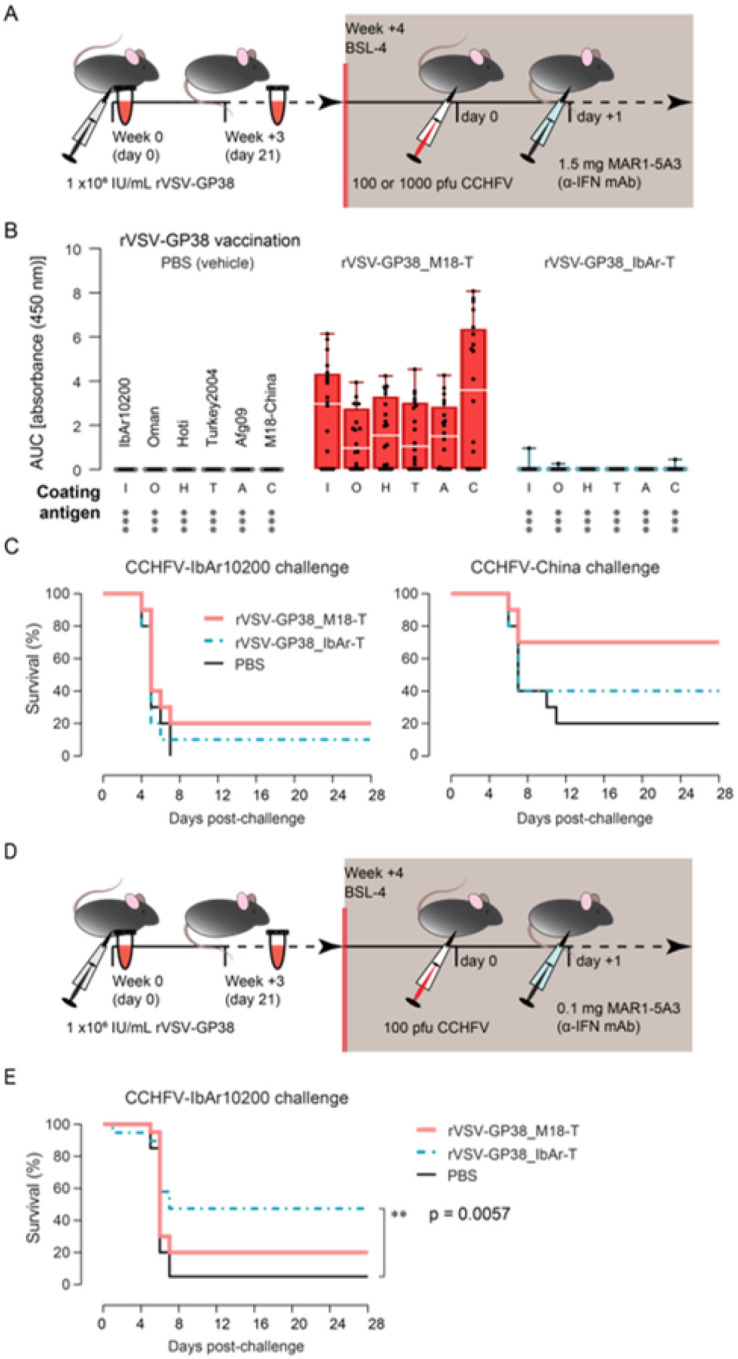
Vaccination with GP38-expressing rVSV provides partial protection against cognate virus **7A** Schematic of mouse vaccination (n=10; rVSV-GP38_M18-China-T, rVSV-GP38_IbAr-T, Vehicle), sera collection schedule, and CCHFV challenge. After vaccination, mice were challenged with 100 pfu CCHFV-IbAr10200 or 1000 pfu CCHFV-China and treated with 1.5 mg of MAR1–5A3 antibody. **7B** rVSV-GP38-vaccinated mouse sera ELISAs: AUC values of each sera group against each rGP38 antigen are individually plotted (n=20 per group). Box-and-whisker plots of AUC values depict range, quartile, and median. AUC values were compared by repeated-measure 2-way ANOVA with Dunnett’s multiple comparisons test where sera response was compared against that of sera from rVSV-GP38_M18-T-vaccinated mice for each coating antigen. **7C** Survival curves of vaccinated mice challenged against CCHFV-IbAr10200 (n=10) or CCHFV-China (n=10). Differences in survival were non-significant by logrank test. **7D** Schematic of mouse vaccination (n=10; rVSV-GP38_M18-China-T, rVSV-GP38_IbAr-T, Vehicle), sera collection schedule, and CCHFV challenge. After vaccination, mice were challenged with 100 pfu CCHFV-IbAr10200 and treated with 0.1 mg of MAR1–5A3 antibody. **7E** Survival curves of vaccinated mice challenged against CCHFV-IbAr10200 (n=10). Survival for each vaccination condition was compared to control (PBS) by logrank test. * p <0.05; ** p <0.01. Non-significant comparisons are unmarked. Exact p-values and descriptive statistics values can be found in **Methods**.

Given the enhanced GP38 expression observed by rVSV-GP38_M18-T (Fig. 6), we selected this virus to vaccinate mice and compared responses to vaccination with rVSV-GP38_IbAr-T. C57BL/6J mice (n = 10) were vaccinated with a single dose (10^6^ IU/mL) of each replication-competent virus or PBS. Three weeks after vaccination, sera were isolated from the immunized mice and evaluated for immunogenicity by ELISA binding to diverse rGP38 antigens (Fig. 7A). The tethered rVSV-GP38_M18-T induced a significantly stronger response in mice compared to mice vaccinated with rVSV-GP38_IbAr-T or PBS-control, with corresponding sera binding to all six rGP38 antigens (Fig. 7B), concordant with our findings with rGP38 immunogens (Figs. 2–3, 5).

Mice were subsequently challenged with 100 pfu of CCHFV-IbAr10200 or -China + 4 weeks post-vaccination and transiently immunosuppressed via administration of 1.5 mg per mouse of MAR1–5A3 (α-IFN mAb) 24 h post-challenge (Fig. 7A). Against CCHFV-IbAr10200 challenge, we observed low protection (≤ 20%) by both rVSV-GP38_M18-T and rVSV-GP38_IbAr-T (Fig. 7B). Against CCHFV-China, we observed greater protection by the cognate (e.g., rVSV-GP18-M18-T) and heterologous VSV vaccines (e.g., rVSV-GP38_IbAr-T) compared to vehicle-control-vaccinated animals, although these differences were not significant (Fig. 7C). Given the overall low protection observed by both rVSV vaccines against CCHFV-IbAr10200, we re-tested protection using a less stringent challenge model by reducing the amount of IFN mAb used for transient immunosuppression. As before, mice were vaccinated with 1×10^6^ IU/mL of rVSV-GP38 vaccine and + 4 weeks post-vaccination challenged with 100 pfu of CCHFV-IbAr10200 followed by transient immunosuppression by treatment with 0.1 mg of α-IFN mAb MAR1–5A3 (Fig. 7D). Similar to the challenge with CCHFV-China, we observed significantly greater protection by the cognate rVSV vaccine (e.g., rVSV-GP38_IbAr-T) relative to vehicle control vaccinated animals and compared to the heterologous rVSV (e.g., rVSV-GP38_M18-T) (Fig. 7E). Overall, these data suggest that the greater humoral immune responses elicited by GP38 derived from the M18-China isolate not sufficient for greater protection in the rVSV vaccine platform, at least in the absence of other CCHFV immunogens (e.g., other GPC sequences).

**7A** Schematic of mouse vaccination (n = 10; rVSV-GP38_M18-China-T, rVSV-GP38_IbAr-T, Vehicle), sera collection schedule, and CCHFV challenge. After vaccination, mice were challenged with 100 pfu CCHFV-IbAr10200 or 1000 pfu CCHFV-China and treated with 1.5 mg of MAR1–5A3 antibody. **7B** rVSV-GP38-vaccinated mouse sera ELISAs: AUC values of each sera group against each rGP38 antigen are individually plotted (n = 20 per group). Box-and-whisker plots of AUC values depict range, quartile, and median. AUC values were compared by repeated-measure 2-way ANOVA with Dunnett’s multiple comparisons test where sera response was compared against that of sera from rVSV-GP38_M18-T-vaccinated mice for each coating antigen. **7C** Survival curves of vaccinated mice challenged against CCHFV-IbAr10200 (n = 10) or CCHFV-China (n = 10). Differences in survival were non-significant by logrank test. **7D** Schematic of mouse vaccination (n = 10; rVSV-GP38_M18-China-T, rVSV-GP38_IbAr-T, Vehicle), sera collection schedule, and CCHFV challenge. After vaccination, mice were challenged with 100 pfu CCHFV-IbAr10200 and treated with 0.1 mg of MAR1–5A3 antibody. **7E** Survival curves of vaccinated mice challenged against CCHFV-IbAr10200 (n = 10). Survival for each vaccination condition was compared to control (PBS) by logrank test. * p < 0.05; ** p < 0.01. Non-significant comparisons are unmarked. Exact p-values and descriptive statistics values can be found in **Methods**.

## DISCUSSION

CCHF is an emerging disease endemic to Europe, Africa, and Asia for which no licensed treatments or vaccines are available. The dearth of CCHFV-specific vaccines has prompted extensive vaccine development efforts^8,45^. However, CCHFV strains are genetically diverse, limiting efficacy, and correlates of protection have been difficult to define due to the multiple vaccine and delivery platforms examined to date and the lack of standardized comparative analyses. Interestingly, the incorporation of GP38 appears crucial for the development of effective CCHFV M-segment-based vaccines^21^. GP38 is integral to viral particle assembly, surface glycoprotein presentation, and pathogenesis, and previous studies investigating GP38-targeting antibodies have been promising^14–19,29^. However, our understanding of GP38 immunogenicity and its contribution to immune protection remains incomplete. Accordingly, we sought to examine its immunological characteristics as a standalone immunogen. We chose six GP38 sequences (IbAr10200, Oman, Hoti, Turkey2004, Afg09, and M18-China) that represent most of the phylogenetic tree, expressed them as recombinant antigens, and evaluated their immunogenicity in mice. Surprisingly, M18-China-rGP38, the most phylogenetically divergent GP38 tested, elicited the broadest humoral immune response in a manner that was independent of the adjuvant or vaccine vector used (Fig. 2, 3, 5, 7). Unfortunately, neither rGP38 subunit (Fig. 5) nor VSV-expressed-GP38 vaccination was significantly protective in a mouse model (Fig. 7).

One possibility for the inadequate protection by our vaccine vectors is due to the choice of adjuvant. Scher et al.’s (2023) rRABV-GP38 vaccine was inactivated and additionally adjuvanted with synthetic monophosphoryl lipid A (MPLA). The resulting serological responses were Th1-skewed, as measured by IgG isotypes^20^. *In vivo* studies with mouse-adapted CCHFV have also shown a Th1-skewed response^46^. In our study, we initially immunized rGP38 with polyIC, which triggers a type I Interferon- (IFN) and Th1-skewed response^47,48^, but subsequently used Addavax, which is similar in formulation to the FDA-approved adjuvant MF59, to elicit both cellular (Th1) and humoral (Th2) immune responses^49,50^. The failure of Addavax to boost vaccine-mediated protection (Fig. 5) suggests that broadening CCHFV vaccines towards a Th2 response may not enhance protection. A second possibility is that humoral immunity elicited by GP38 is insufficient for protection. Several CCHFV experimental vaccine studies have identified roles for cell-mediated and humoral immunity, with some instances of neutralizing antibody production^22,51,52^. However, other studies have shown that the humoral response, while important, is not consistently correlated with protection and is inadequate for vaccine-mediated protection, with a more prominent role for T-cell responses following vaccination^11,23,31,53–56^. Notably, immunization with Hoti-rGP38 adjuvanted with both MPLA and Addavax did not protect against CCHFV-IbAr10200 in mice^24^. Although vaccination with rGP38 of the M18-China strain resulted in enhanced humoral immune responses, our analysis did not consider potential differences in cell-mediated T-cell responses that could drive differences in protection against infection. In fact, recent reports suggest that T-cell responses to GP38 may be CCHFV-isolate-specific^21^. Future work is needed to comprehensively evaluate the humoral and cell-mediated responses following vaccination with GP38 from diverse CCHFV isolates across distinct vaccine platforms and alternative adjuvants.

We uncovered isolate-dependent GP38 immunogenicity differences with interesting implications for future CCHFV studies on pathogenesis, structure, and therapeutics. Our recent work has discovered a novel role for GP38 as a viral toxin that causes barrier dysfunction and vascular leak *in vivo*^19^. However, this work was conducted using the CCHFV-IbAr10200 strain only. Based on the results reported here, we anticipate that additional investigations of GP38-triggered vascular leak employing diverse rGP38 antigens and authentic CCHFV isolates could reveal isolate-specific differences in vascular leak that drive pathogenesis. Further, almost all of the structural information currently available for components of the CCHFV glycoprotein complex, including GP38, has been limited to the CCHFV IbAr10200 strain^14,16,57^. A recently solved structure of GP38 from the CCHFV Hoti strain points to some (relatively minor) differences between the Ibar10200 and Hoti GP proteins^29^. Although these differences did not appear to impact binding of protective GP38-targeting antibodies, they have not been evaluated in regard to immunological responses. GP38 from CCHFV Hoti has an approximate 16% amino acid sequence identity difference with GP38 from CCHFV IbAr10200, versus an approximate 26% difference between GP38 from M18-China and IbAr10200^18^. This raises the question of whether the greater sequence difference for M18-China translates to larger structural differences in GP38, or to overall changes in the structure of the stable complex formed between GP38, Gn, and Gc. Even slight modifications to the interactions within the glycoprotein complex could mask, or unmask, epitopes that contribute to greater immunological responses. As demonstrated through our chimeric rGP38 antigens (Fig. 4), substitutions in less conserved GP38 sequences greatly affected the subsequent humoral response.

To our knowledge, no reports exist comparing the immunogenicity and protective potential of GP38 proteins from divergent CCHFV clades. More generally, few evaluations of the heterologous efficacy of CCHFV-specific vaccines and therapeutics have been described. The wealth of data pertaining to the characterization of broad antibody responses elicited by GP38 of the M18-China strain described in this study should facilitate efforts to develop more broadly effective vaccines and antivirals. While rGP38-only subunit and rVSV vaccinations did not result in improved survival, incorporating M18-China GP38 antigens into additional vaccine platforms that have shown promising protection could enhance the efficacy of these vaccine candidates. Moreover, vaccines that utilize CCHFV-GP38 in combination with other immunogenic proteins, such as NP, Gc, or the complete M-segment, could benefit from investigating whether incorporating the GP38 sequence from M18-China could prompt better, more broad immunological responses and even cross-protection against diverse CCHFV isolates. Indeed, recent work by Karaaslan et al. (2024) demonstrated decreased morbidity in mice after vaccinating with a combination of Hoti-NP and -GP38 subunits relative to -NP alone; both regimens were capable of conferring significant protection against heterologous CCHFV-IbAr10200 challenge in mice^24^. We postulate that the addition of M18-China-rGP38 could provide greater protection, since Hoti-rGP38 not only induces poor cross-reactive humoral immune responses, but also poorer humoral responses overall relative to other isolates of rGP38 (Figs. 3, 5). Our findings indicate that the systematic investigation of isolate- and strain-dependent differences in other immunogenic proteins, such as Gc and NP, could be fruitful for the further development of potently and broadly protective CCHFV vaccines.

## METHODS

### Ethics statements

All animal studies were performed at facilities accredited by the Association for Assessment and Accreditation of Laboratory Animal Care, International (AAALAC). Experiments were approved by their respective Institutional Animal Care and Use Committees (IACUC) in compliance with the Animal Welfare Act, PHS policy, other applicable state and federal statutes and regulations, and adhere to the principles stated in the Guide for the Care and Use of Laboratory Animals, National Research Council, 2013. Non-challenge murine immunization studies were performed at Albert Einstein College of Medicine (protocol no: 00001176). Murine challenge and vaccination studies were conducted at the United States Army Medical Research Institute of Infectious Diseases (USAMRIID). Humane endpoints were utilized during these studies and mice that were moribund, according to an endpoint score sheet and in line with IACUC-approved criteria, were humanely euthanized.

### Cells

Vero cells (American Type Culture Collection [ATCC]) were cultured in Dulbecco's Modified Eagle Medium (DMEM; Thermo Fisher Scientific [TFS]) supplemented with 2% heat-inactivated fetal bovine serum (Bio-Techne/Gemini-Bio), 1% penicillin-streptomycin (P/S; TFS), and 1% GlutaMAX (TFS). 293FT cells (TFS) and BSR-T7 cells were cultured in DMEM supplemented with 10% FBS, 1% P/S, and 1% GlutaMAX. BSRT7 cells (RRID: CVCL_RW96), a spontaneously immortalized cell line isolated from the kidney of a Golden hamster, stably expresses T7 RNA polymerase and were a kind gift from K.-K. Conzelmann. These cells were not authenticated following gifting. Adherent cells were maintained in a humidified 37 °C incubator supplied with 5% CO_2_. Freestyle 293-F cells (Invitrogen/TFS) were maintained in Freestyle 293 expression media (TFS) with 1% P/S at 37 °C, with 8% CO_2_ in a humidified shaking incubator.

### Virus stocks

The authentic CCHFV isolates CCHFV-IbAr10200, CCHFV-Afg09–2990 (labeled as ‘CCHFV-Afg09’), and CCHFV-HY13-China (labeled as ‘CCHFV-China’) were used in this study.

### Generation of GP38 phylogenetic tree

The following CCHFV isolates (and GenBank accession numbers) were used in this study as reference sequences: IbAr10200 (NC005300), Oman-199809166 (KR864901; Oman), Kosova-Hoti (EU037902; Hoti), 200406546-Turkey (KR864902; Turkey2004), Afg09–2990 (HM452306; Afg09), and 79121M18 (GU477493; M18-China).

Two searches for GP38 amino acid sequences were performed. The first search was performed in GenBank with the following parameters on October 26, 2023: (“Orthonairovirus haemorrhagiae” [Organism] AND (“M”[All Fields] OR “glycoprotein”[All Fields])) AND “complete”[All Fields] AND (“5000” [SLEN] : “6000”[SLEN]). This search reported 295 CCHFV complete M segments. To identify the approximate boundaries of the GP38 protein within the M segments, we blasted each of the six CCHFV reference GP38 proteins (IbAr10200, Oman, Hoti, Turkey, Afg09, and M18-China) against the M segments. We accounted for potential misaligned regions at the N- and C-terminus by setting up the initial GP38 boundaries 20 amino acid positions before and after the alignment start and end positions. A second search was conducted for “Bunyavirus” in the Viral Bioinformatics Resource center (ViPR-BRC) in December 2023^58^. Using this public repository, we first downloaded all protein sequences (independent of the segment they belong) for any annotated CCHFV (4,321 proteins). Next, we blasted each of the six CCHFV reference GP38 proteins (IbAr10200, Oman, Hoti, Turkey, Afg09, and M18-China) against all sequences downloaded from ViPR-BRC, requesting a minimum coverage of 90% over the length of the query proteins^59^. We identified 281 hits with an e-value ≤ 1×10^−120^. As described above, we used the blast output to identify the initial GP38 boundaries (±20 residues).

Comparing both search approaches using the GenBank identifier showed a good overlap between both GP38 datasets (272 proteins in common). The differences were either due to recently published sequences considered in GenBank but not in ViPR-BRC, or incomplete M segments that included GP38 proteins. Altogether, the combined dataset contained 303 CCHFV GP38 proteins. To identify the exact GP38 boundaries, we performed a multiple sequence alignment (MSA) with Clustal Omega and used Jalview to trim all the N- and C-term segments that were not aligned to the six reference GP38 proteins^60,61^. At this stage, we removed one GP38 protein (GenBank ID: KX238957) that was missing the N-term.

We performed a second MSA with Clustal Omega using the final dataset of 302 GP38 protein sequences trimmed to match the length of the reference GP38s. Next, we loaded the MSA in JalView and generated a tree using the average distance mode^60^. The tree was subsequently visualized using iTOL, adding additional information to the tree (e.g. the region where the virus was isolated, Figure 1)^62^.

### Expression and purification of recombinant CCHFV GP38

Recombinant CCHFV GP38 (rGP38) proteins were expressed and purified as previously reported for the following isolates: IbAr10200, Oman, Hoti, Turkey, Afg09, and M18-China^16,18^. Briefly, the MLD-GP38 genes for each isolate were cloned into the pαH eukaryotic expression vector with C-terminal purification tags (human rhinovirus [HRV] 3C protease cleavage site, an 8x His tag, and a Twin-Strep tag). To aid in cleavage of MLD from GP38 during transfection, a pCDNA3.1 plasmid encoding furin was co-transfected at a 1:9 furin:GP38 plasmid ratio. Using polyethyleneimine (PEI), the two plasmids were transiently transfected into FreeStyle 293-F cells followed by the addition of 5 μM kifunensine for uniform high-mannose glycosylation. The secreted GP38 was harvested from the media, followed by affinity purification with Ni-NTA resin (TFS HisPur^™^ Ni-NTA Resin). The GP38 Ni-NTA eluent was treated with HRV 3C protease (in-house) and further purified by size-exclusion chromatography (SEC) with a HiLoad 16/600 Superdex 200 column (GE Healthcare Biosciences) in 1X PBS. rGP38 proteins were concentrated, aliquoted, flash frozen in liquid nitrogen, and stored at −80°C.

### Chimeric Oman-M18-China rGP38 design, expression, and purification

5x gene fragments were ordered and produced by Twist Bioscience and encoded for CCHFV-M18-China MLD and CCHFV-M18-China GP38 with the relevant Oman strain sequence substitutions. The substituted amino acids are indicated in brackets: ONTer GP38 [248–310], OVar1 GP38 [321–342], OVar2 GP38 [376–395], ON-v1–2 [248–310, 321–342, 376–395]. The original MLD-GP38 sequence was excised from the pαH expression by restriction enzymatic digestion and the MLD and chimeric GP38 sequences were inserted by NEBuilder Hi-Fi DNA assembly (NEB) per manufacturer’s instructions. The resulting plasmids were sequenced via whole plasmid sequencing by Plasmidsaurus using Oxford Nanopore Technology with custom analysis and annotation. As before, a pCDNA3.1 plasmid encoding furin was co-transfected with each chimeric GP38 plasmid at a 1:10 furin:GP38 mass ratio into FreeStyle 293-F cells using PEI. After 6 days, secreted GP38 was harvested by Ni-NTA affinity purification (as described above) and concentrated, treated with HRV 3C (Millipore Sigma) overnight at 4°C, and further purified by SEC at the Einstein Macromolecular Therapeutics Development Facility into 1X PBS. Chimeric proteins were concentrated, aliquoted, flash frozen in liquid nitrogen, and stored at −80°C.

### Generation of tecVLPs

The amino acid sequences for the IbAr10200 were derived from GenBank M-segment sequences with an accession number NC005300. Transcription- and entry-competent virus-like particles (tecVLPs) bearing CCHFV glycoproteins were generated as previously described^17,18,63^. Briefly, BSR-T7 cells were transfected with five plasmids separately encoding the CCHFV nucleoprotein (NP), glycoprotein complex (GPC), polymerase (L), T7 polymerase, and a Nano-Glo luciferase minigenome in the absence of P/S. 15 h post-transfection, transfection media was replaced on cells with P/S-containing DMEM growth media. 60 h post-transfection, tecVLP-containing supernatants were collected, clarified by low-speed centrifugation, and pelleted by ultracentrifugation (Beckman SW28 rotor, 25,000 rpm/112,400 *g*) for 2.5 h. Pelleted tecVLPs resuspended in DMEM overnight before aliquoting and storage at −80°C.

### Conformational ELISAs of rGP38 and chimeric variant antigens

ELISAs were run in technical duplicates. Flat bottom, high bind, half-area 96-well plates (Corning) were coated with 75 ng of each rGP38 antigen in 1X PBS overnight at 4°C (~14–16 h). The following day, the plate was washed 3x with 1X PBS before being blocked with 5% nonfat dry milk (BioRad) in 1X PBS (5% milk) for 3 h at 37°C. 3-fold serial dilutions were made for ADI-58048 and ADI-63530 starting at either 100 or 30 nM in 3% bovine serum albumin (BSA; Fisher) in 1X PBS (PBSA) and incubated on the plate for 1.5 h at 37°C. After 3x washes with 3% milk in 1X PBS, the plate was subsequently incubated with an anti-human IgG horseradish peroxidase (HRP) (Millipore Sigma, cat. AP112P) secondary in 3% milk for 1 h at 37°C. The plate was then washed 5x with 1X PBS, and the ELISA was developed at room temperature (RT) in the dark with 1-Step TMB Ultra Substrate (TFS) before being neutralized with 0.5 M H_2_SO_4_. Binding was quantified using Cytation 5 cell imaging multimode reader (Agilent/BioTek, v3.1.2) to measure absorbance at 450 nm.

### In vivo rGP38 immunization studies

6–8-week-old female C57BL/6J mice were purchased from the Jackson Laboratory (JAX: 000664). Mice were immunized with 10 μg of indicated rGP38 immunogen or an equal volume of phosphate-buffered saline (PBS) adjuvanted with 50 μg of poly(I:C) (High Molecular Weight) VacciGrade (InvivoGen) and boosted three weeks after in the same fashion. Constructs and adjuvant were each diluted in endotoxin-free PBS (Millipore) to a total volume of 100 μL each before they were mixed to obtain a total of 200 μL per mouse. 200 μL of immunogen + adjuvant was delivered by i.p. injection for each mouse. Mice were bled by submandibular bleeding 1–7 days before prime and boost. Blood was additionally collected post-boost at the indicated days in the pertinent figures. Sera were isolated from whole blood by allowing the blood to coagulate for 1 h at RT and separated by microcentrifugation (10000 *g*, 10 min). Sera were aliquoted and stored at −80 °C for subsequent use in ELISA and tecVLP neutralization assays.

### Serum ELISAs from rGP38 immunizations

Serum ELISAs were performed as described for the conformational rGP38 ELISAs with modifications in the primary and secondary antibody incubation steps. After blocking the GP38-coated plate, 3-fold serial dilutions were made for sera samples starting at a 1:50 dilution in 3% PBSA and incubated on the plate for 1.5 h at 37°C. The plate was subsequently incubated with an anti-mouse IgG-HRP secondary (Jackson Immunolabs, cat. 115-035-003) secondary in 3% milk for 1 h at 37°C. Development and readout were performed as described for conformational rGP38 ELISAs.

### Sera tecVLP neutralization

TecVLPs bearing IbAr10200 glycoproteins were titered after generation, and an empirical dilution was chosen for neutralization assays such that the maximum luminescence signal was two orders of magnitude higher than that of background. Neutralization against CCHFV IbAr10200 tecVLPs were assessed in Vero cells, as described previously^17,18,64^. Vero cells were seeded in 96-well flat, clear bottom, white cell culture plates (Corning) at 18,000 cells per well 24 h before infection. 3-fold serial dilutions were performed for each sera sample starting at 1:150. TecVLPs were then incubated with the antibodies for 1 h at 4°C before the antibody/tecVLP mixtures were added onto the cells. Infection was allowed to proceed in a humidified 37°C incubator supplied with 5% CO_2_ for 14–16 h. The infection media was then dumped, cells were washed once with PBS, and luminescence signal was developed using the Nano-Glo luciferase assay system (Promega) per manufacturer’s instructions. Infectivity was quantified by luminescence signal using Cytation 5 cell imaging multimode reader (Biotek/Agilent, v3.1.2).

### Sera-rGP38 immunoassay by Luminex MAGPIX

rGP38 from each CCHFV isolate was coupled to the indicated Luminex MagPlex-C Microspheres (regions): IbAr10200 (015), Oman (018), Hoti (021), Turkey (027), Afg09 (029), M18-China (033) at a concentration of 3 μg protein / 1×106 microspheres using the xMAP Antibody Coupling Kit (Luminex) per manufacturer instructions. Coupled microspheres were counted, aliquoted, and stored in 1% PBSA at 4°C until further use. The following immunoassay was adapted from Chapter 4.3.3 in the xMAP Cookbook, 5th edition (Luminex). rGP38-coupled microspheres for each isolate and MagPlex RP1 Monitor microspheres were vortexed and pooled together in 1% PBSA at a density of 50 microspheres per μL. The microsphere mixture was vortexed again and 50 μL of the mixture was added to a 96-well, black, flat bottom microplate (Greiner Bio-one). The plate was incubated at RT for 30 min at 400 rpm. The microspheres were settled by a magnetic separator (Luminex), supernatant decanted, and sera samples (diluted 1:50) were added to the plate in technical duplicates, and sera were incubated with the microspheres overnight, at 4°C. The following day, the microspheres were washed 3x with 1% PBSA and incubated with anti-mouse IgG-PE (Southern Biotech; cat. 1036–09) at a dilution of 1 μg/mL for 30 min, at RT, rocking at 400 rpm. The plate was washed an additional 3x with 1% PBSA before being re-suspended in Luminex running buffer. The plate was read by the Luminex MAGPIX system at the Einstein SSR-CFAR-HIV Mucosal Immunity Core.

### Animal challenge experiments

4–6-week-old female C57BL/6J mice were purchased from the Jackson Laboratory (JAX: 000664). For rGP38 vaccinations, mice were immunized with 10 μg of indicated rGP38 immunogen or an equal volume of phosphate-buffered saline (PBS) adjuvanted with an equal volume of AddaVax (100 uL; InvivoGen) and boosted three and six weeks after in the same fashion. Constructs were diluted in endotoxin-free PBS (Millipore). 200 μL of immunogen + adjuvant was delivered by i.p. injection for each mouse. Mice were bled by submandibular bleeding on days 0, 21, 42, and 56 prior to vaccination. Sera were isolated from whole blood by allowing the blood to coagulate for 1 h at RT and separated by microcentrifugation (10000 g, 10 min). Sera were aliquoted and stored at −80°C for subsequent use in ELISA assays. 9 weeks post-vaccination, mice were challenged with 100 PFU pfu of CCHFV-IbAr10200, CCHFV-Afg09, or CCHFV-HY13 China by the i.p. route. 24 h post-challenge, mice were transiently immunosuppressed by treatment with 1.5 mg/mouse of mAb-5A3 (Leinco Technologies Inc.) via the i.p. route. Mice were monitored daily for weight changes, clinical score, and survival. Mice were scored on a 4-point grading scale; 1= decreased grooming and ruffled fur, 2= subdued behavior when un-stimulated, 3= lethargy, hunched posture, and subdued behavior even when stimulated, and 4= bleeding, unresponsiveness, severe weakness, or inability to walk. All mice scoring a 4 were considered moribund and were euthanized based on IACUC-approved criteria. Daily observations were increased to a minimum of twice daily while mice were exhibiting a clinical score of 3.

For rVSV vaccination experiments, mice were immunized with 10^6^ infectious units /mL of the indicated rVSV-GP38 vaccine or an equal volume of endotoxin-free PBS. 200 μL of immunogen + adjuvant was delivered by i.p. injection for each mouse. Mice were bled by submandibular bleeding on days 0 and 21. Sera were isolated from whole blood and stored as described above. 4 weeks post-vaccination, mice were challenged with 100 pfu of CCHFV-IbAr10200 or CCHFV-China by the i.p. route. 24 h post-challenge, mice were transiently immunosuppressed by treatment with 1.5 or 0.1 mg/mouse of mAb-5A3 (Leinco Technologies Inc.) via the i.p. route. Mice were monitored daily for weight changes, clinical score, and survival as described above.

### Serum ELISAs from rGP38 vaccination

For heterologous ELISAs, n=10 mice from each group were pooled from the pre-vaccination day 0 samples to generate two separate pools per group. Sera from day 56 bleeds were run as individual samples for all groups. For homotypic ELISAs, n=10 mice from each group were pooled for all time points to generate two separate pools per group for all groups. High bind half-area ELISA plates (Corning) were coated overnight (~18 h) at 4°C with 75 ng rGP38 protein diluted in PBS per well. The following day, plates were blocked with 5% skim milk (BD Biosciences) diluted in PBS containing 0.05% Tween-20 (PBST) for 2 h at 37°C. Sera samples were diluted 1:10 with subsequent 3-fold dilutions (dilution range 1:10 to 1:7290) in blocking buffer and plates were loaded with dilutions in duplicate. Plates were incubated at RT for 2 h, washed three times with PBST, and then incubated with HRP conjugated goat anti-mouse (Jackson ImmunoResearch) diluted in blocking buffer for 1 h at RT. After incubation, plates were washed three times with PBST and then developed with TMB substrate (TFS). Reaction was stopped using 0.16 M H_2_SO_4_ and absorbance was read at 450 nm wavelength, detected using a SpectraMax (Molecular Biosciences) microplate reader. A cutoff value was determined based on the average absorbance of a naïve control starting dilution plus 3 standard deviations. Only sample dilutions whose average were above this cut-off were registered as a positive signal.

### Generation of rVSV-GP38 viruses

The starting plasmid used for cloning encoded for the VSV antigenome and an additional eGFP reporter gene as the first separate transcriptional unit, but it lacked the transcriptional unit for VSV-G. This plasmid was modified by replacing the eGFP sequence with the CCHFV-MLD and -GP38 from the IbAr10200 or M18-China strains by NEBuilder HiFi DNA assembly. 70 amino acids from the C-terminal end of VSV-G were included for the tethered GP38 viruses. VSV-G was subsequently reinserted by NEBuilder HiFi DNA assembly to generate the genomic plasmids of rVSV-eGFP and the corresponding rVSV-GP38 viruses (rVSV-GP38_M18, rVSV-GP38_M18-T, VSV-GP38_IbAr, rVSV-GP38_IbAr-T). Plasmid-based rescue of rVSV was performed as previously described^42–44^. Briefly, for each rVSV, 293FT cells were transfected with the modified VSV antigenome plasmid with 6x helper plasmids that express T7 polymerase, VSV-N, -P, -M, -G, and -L proteins using PEI. Supernatants from the transfected cells were transferred to Vero cells 2- and 3-days post-transfection and propagated for an additional 2–3 days. Virus-containing supernatant from Vero cells were plaque purified. RNA was isolated from 3x plaques for each virus, converted to cDNA by RT-PCR, amplified by PCR, and sequenced by Plasmidsaurus linear/PCR sequencing using Oxford Nanopore Technology with custom analysis and annotation for MLD-GP38 insertion and sequence verification. Sequenced rVSV-GP38 plaques (Passage 0) were passaged twice for viral expansion. Viral supernatants from the 2nd passage were clarified by low-speed centrifugation before concentration by ultracentrifugation (Beckman SW28 rotor, 20,000 rpm/72,000 *g*). Viral pellets (passage 2; P2) were re-suspended in 1X PBS, aliquoted, and frozen.

### Titering rVSV-GP38 viruses

Vero cells were seeded in 96-well flat, clear bottom, cell culture plates (Corning) at 20,000 cells per well 24 h before infection. 10-fold serial dilutions were made for each rVSV from 10^−1^ to 10^−11^. Cells were infected for 1 h at 37°C with each dilution in triplicate, and infection was stopped with 20 mM NH_4_Cl solution. Cells were further incubated in a humidified 37°C incubator supplied with 5% CO_2_ for 10 h before immunostaining for VSV-M. Infected cells were fixed with 4% paraformaldehyde (TFS) for 10 min. Cells were next washed with 1X PBS, permeabilized with 0.1% Triton X-100 (Sigma) for 15 min, and blocked for 30 min with 10% FBS in 1X PBS. VSV-M was detected by incubating cells with an in-house anti-VSV-M mAb (1.5 μg/mL, mouse) for 1 h followed by anti-mouse IgG (H+L) AF488 (TFS, cat. A11029). Cells were washed 3x with 1X PBS between steps. Nuclei were detected by Hoescht 33342, trihydrochloride trihydrate (TFS, cat. H3570). Titers were quantified by counting the number of cells positive for VSV-M.

### Qualifying GP38 expression in rVSV-GP38 viruses by western blot

Equal-volume amounts of P2 rVSV-eGFP and rVSV-GP38 virus pellets were denatured at 95°C for 5 min in 4X Bolt LDS NuPAGE sample buffer (TFS). Equal-volume amounts of total virus (pre-concentrated passage 1 virus stocks) were similarly denatured. Denatured proteins were loaded in Bolt bis-tris miniprotein gels, 4–12% (TFS) and run at a constant 130V at RT for 75 min. Proteins were transferred to a 0.2 μm nitrocellulose membrane (constant 400 mA, 4°C, 1 h) with Bolt transfer buffer (TFS). Membranes were next blocked with 5% milk for 1 h at RT. CCHFV-GP38 and VSV-M were respectively detected by incubating the membrane overnight (~14 h) with a previously isolated anti-GP38 human mAb ADI-46152^17^(0.5 μg/mL) and 23H12, an anti-VSV-M mAb (0.75 μg/mL, mouse) gifted by Wendy Maury from Douglas Lyles, followed by anti-human IRDye 800CW (Licor, cat. 926–32232) and anti-mouse IgG (H+L) AF680 (TFS, cat. A21057). The membrane was washed 3x with 1X PBS after blocking and 3x with 0.1% Tween 20 in 1X PBS (PBS-T) after each antibody incubation step. Western blots were imaged on an iBrightFL1500 imager (Invitrogen/TFS, software v1.8.1).

### Qualifying GP38 expression in rVSV-GP38 infected cells by immunofluorescence

Vero cells were seeded on 15 mm glass coverslips coated with fibronectin (1:20) in 1X PBS in 24-well clear tissue-culture treated plates (Corning) at 180,000 cells per well 24 h before infection. Vero cells were infected by rVSV-GP38 viruses at a MOI = 1 or 0.1 for 1 h at 37°C, and infection was stopped with 20 mM NH_4_Cl. Cells were incubated for an additional 12 h in a humidified 37°C incubator supplied with 5% CO_2_ before immunostaining for intracellular and surface GP38. Intracellular staining for GP38 was similarly as described for tittering. GP38 was detected by incubating cells with human ADI-46152 (0.5 μg/mL) for 1 h followed by anti-human IgG (H+L) AF594 (TFS, cat. A11014). Surface staining for GP38 necessitated that ADI-46152 was added prior to fixation. Thus, cells were blocked in 10% FBS in 1X PBS at 4°C for 30 min before they were incubated with ADI-46152 (0.5 μg/mL) for 1 h at 4°C. Cells were then fixed with 4% PFA and incubated with anti-human IgG (H+L) AF594 for 1 h at RT. Cells were washed 3x with 1X PBS between steps. Nuclei for both immunostaining conditions were detected by Hoescht 33342, trihydrochloride trihydrate. All coverslips were mounted on glass slides with ProLong Gold Antifade (TFS) and allowed to cure overnight at RT. Cells were examined using an Axio Observer Z1 wide-field epifluorescence microscope (Zeiss Inc., ZEN Imaging software, ZEN2 blue edition) with a 63x objective.

### Statistical and reproducibility

Replicates are indicated in the respective figures (N = experimental replicates; n = total technical replicates). All statistical tests, curve-fittings, and AUC calculations were performed in Prism (GraphPad, v10.0.3 [275]). ELISA and neutralization data were fitted with a nonlinear regression model (variable slope, 4 parameters). AUC values were computed for each ELISA curve (Baseline Y=0, Ignore peaks <10% of the distance from min to max Y).

In Figure 2, AUC values were calculated from ELISA data (n=2). **Supplementary Table 1** depicts associated descriptive statistics.

In Figure 3, MFI values (n=4) (Figure 3B) were averaged and plotted for each mouse within each group (n=6 / group). **Supplementary Table 2** depicts associated descriptive statistics.

In Figure 4, AUC values (Figure 4D) were calculated from ELISA data (n=2) (**Supplementary Figure 3**) for each mouse sera sample within each group (n=5 / group) and graphed as a box-and-whisker plot with median depicted. AUC values were log_10_-transformed to fulfill normality assumptions before a repeated measure two-way repeated-measures ANOVA (Geisser-Greenhouse correction) with Dunnett’s test for multiple comparisons (relative to M18-China-rGP38-immunzied sera) was performed with a family-wide α=0.05. Lognormality was assessed by Shapiro-Wilk test (passed) and QQ plot. The two-way ANOVA model was subsequently evaluated by residual, homoscedasticity, and QQ plots. **Supplementary Table 3–4** depicts associated descriptive statistics and exact p-values, respectively.

In Figure 5, AUC values (Figure 5B) were calculated from ELISA data (n=2) (**Supplementary Figure 4**) for each mouse sera sample within each group (n=20 / group) and graphed as a box-and-whisker plot with median depicted. A two-way repeated-measures ANOVA (Geisser-Greenhouse correction) with Dunnett’s test for multiple comparisons (relative to M18-China-rGP38-vaccinated sera) was performed with a family-wide α=0.05. Data normality was assessed by Shapiro-Wilk test (passed) and QQ plot. The two-way ANOVA model was subsequently evaluated by residual, homoscedasticity, and QQ plots. **Supplementary Tables 5–6** depicts associated descriptive statistics and exact p-values, respectively. Descriptive statistics for Figure 5C is shown in **Supplementary Table 7**, where each value in n is pooled sera from 5 mice. Survival curves of rGP38-vaccinated conditions were compared against PBS-control and each other by logrank tests with a Bonferroni corrected α =0.05/3 = 0.0167 in Figure 5E. Associated p-values are shown in **Supplementary Table 8**.

Descriptive statistics for Figure 6B (rVSV titers) are shown in **Supplementary Table 9**.

As seen above, for AUC values in Figure 7B, a two-way repeated-measures ANOVA (Geisser-Greenhouse correction) with Dunnett’s test for multiple comparisons (relative to rVSV-GP38_M18-T-vaccinated sera) were performed with a family-wide α=0.05 after data normality was assessed by Shapiro-Wilk test (passed) and QQ plot. **Supplementary Tables 10–11** show descriptive statistics and p-values. Survival curves shown in Figures 7C, 7E were compared against each other by logrank tests with a Bonferroni corrected α=0.05/3 = 0.0167. Associated p-values are shown in **Supplementary Table 12**.

## Supplementary Files

This is a list of supplementary files associated with this preprint. Click to download.

- GP38breadthSUPPSpringer15May2026.docx
- GP38breadthSUPP2SpringerGelblots18May2026.docx

## Data Availability

All relevant data generated or analyzed during this study are included within this published article and its supplementary information files. Relevant raw data associated with this published article can be found at: https://doi.org/10.6084/m9.figshare.c.8477811.
